# Factors Influencing Retention at Their First Hospital Among New Graduate Nurses in South Korea

**DOI:** 10.3390/healthcare14030314

**Published:** 2026-01-27

**Authors:** Yujin Jeong, Hyo-Jeong Yoon

**Affiliations:** 1 Department of Nursing, Yeungnam University College, Daegu 42415, Republic of Korea; yjjeong@ync.ac.kr; 2 College of Nursing, Dankook University, Cheonan 31116, Republic of Korea

**Keywords:** nurses, retention, personnel turnover, job satisfaction, transition model

## Abstract

**Background/Objectives:** Early turnover among new graduate nurses remains challenging in South Korea. This study examined how socialisation factors—based on Scott et al.’s transition model and Herzberg’s motivation-hygiene theory—are associated with early retention at the first hospital of employment among new graduate nurses. **Methods:** This retrospective cross-sectional study analysed secondary data from the Graduate Occupational Mobility Survey (GOMS), a nationally representative dataset of college and university graduates in Korea, collected using a stratified multi-stage sampling method. The study included 602 new graduate nurses from the 2017–2019 datasets who had worked as nurses at their first hospital of employment. Anticipatory socialisation factors included personal and educational characteristics. Organisational socialisation factors referred to workplace-related characteristics of the first hospital, including motivational factors and hygiene factors. The outcome variable was early retention. Multiple logistic regression analyses were performed to identify factors associated with early retention. **Results:** A total of 68.6% of nurses remained in their first hospital employment. Retention was more likely among nurses whose high school, nursing school, and first hospital were in the same region (*p* = 0.019), those employed in Seoul (*p* < 0.001), and those working in larger hospitals (*p* < 0.001). Retention was also associated with satisfaction with autonomy and authority (*p* = 0.013). Conversely, lower retention was observed among nurses who were dissatisfied with interpersonal relationships (*p* < 0.001) and those who reported satisfaction with growth opportunities (*p* < 0.001). **Conclusions:** Targeted strategies that support new graduate nurses during their transition are essential. Aligning education-to-employment regions and strengthening workplace conditions may enhance early retention.

## 1. Introduction

The shortage of nurses essential for ensuring patient safety and delivering high-quality care is a global issue. South Korea ranks among the top countries in the Socio-Demographic Index; however, the number of nurses per 10,000 people is only 52.6, less than half of the average of 114.9 in these leading countries [1]. In South Korea, although the number of new graduate nurses is increasing [2], the persistent shortage of nurses due to high turnover rates remains a significant issue. Nurse turnover continues contributing to this shortage, resulting in hospitals with inadequate nurse staffing. Consequently, patients in these hospitals are more likely to experience higher mortality rates, increased readmission rates, and longer hospital stays [3].

Notably, nurse turnover in South Korea is particularly high within the first one to two years of hospital employment [4,5]. The first year after graduation is critical for new graduate nurses as they develop professionally and adapt to the clinical environment. During this transition, they often face various challenges [6]. New graduate nurses experience difficulties in hospital settings, including adapting to new roles, coping with excessive workloads, and managing interpersonal relationships [7]. Previous studies have reported that new graduate nurses’ turnover intention increases when they experience limited support, poor work environments, and difficulties adapting to professional roles [8]. Additionally, previous studies have highlighted the impact of hospital size, location, work intensity, and the overall work environment on turnover rates [5,9]. Job satisfaction and dissatisfaction, which are influenced by multiple factors according to Herzberg et al.’s motivation-hygiene theory [10], may be related to turnover. Informed by this perspective, the present study considered Herzberg et al.’s theory in identifying organisational factors relevant to new graduate nurses’ retention.

Nursing education has also been recognised as a key factor influencing job retention [11]. The successful transition of nurses within the first two years after graduation should begin during undergraduate education, including clinical training [12,13]. Successfully completing nursing education programmes and gaining diverse practical experiences such as internships are crucial for equipping new graduate nurses with the competencies required in clinical settings, ultimately playing a significant role in their long-term career retention. Meanwhile, nurses tend to prefer working at hospitals in regions where they grew up or were trained [14], supporting long-term nurse retention.

Previous studies mostly focused on turnover intention rather than actual turnover and have typically been conducted in geographically restricted settings or within only a few institutions [4,15,16]. Moreover, although educational preparation, geographical background and organisational elements, including hospital characteristics and motivation-hygiene factors, have been identified as relevant, few studies have examined these domains concurrently using nationally representative data [17]. These gaps highlight the need for research that integrates multiple levels of socialisation factors and investigates their relationship with actual early retention among new graduate nurses.

To address these gaps, the present study draws on Scott et al.’s transition model [6] and Herzberg et al.’s motivation-hygiene theory [10,18] to provide a theoretically grounded framework for examining these socialisation influences. Scott’s model distinguishes anticipatory socialisation—formed through educational preparation and personal background—from organisational socialisation, which encompasses the actual experiences and adaptation processes that occur after entering the hospital [6]. In this study, the organisational socialisation stage was further informed by Herzberg’s framework to guide the selection of workplace-related variables associated with job satisfaction and dissatisfaction in the first hospital of employment [10,18]. Herzberg’s theory complements this perspective by differentiating between motivators that enhance job satisfaction and hygiene factors that prevent dissatisfaction [10,18].

Accordingly, this study aims to (a) describe early job retention among new graduate nurses in South Korea approximately 18 months after graduation and (b) analyse the associations of anticipatory socialisation factors—such as educational preparation and geographical background—and organisational socialisation factors, including motivation and hygiene factors, with early retention in the first hospital setting.

## 2. Conceptualisation and Theoretical Framework

This study applied Scott et al.’s [6] transition model and Herzberg’s [10,18] motivation-hygiene theory to explore the adaptation process of new graduate nurses within healthcare organisations. Scott et al.’s [6] transition model includes three key stages of workplace adjustment: anticipatory socialisation, organisational socialisation, and socialisation outcomes. Socialisation outcomes represent the results of the socialisation process, which may be either positive or negative; in this study, the outcome of interest was the early retention of new graduate nurses.

Anticipatory socialisation refers to expectations and perceptions formed about the first workplace, influenced by personal and educational experiences such as age, sex, and academic background. Organisational socialisation refers to the actual experiences encountered after entering the workplace. During this stage, new graduate nurses adjust to the clinical environment. In this context, workplace factors were considered relevant for understanding variations in retention. In line with these conceptual dimensions, Lee et al. [19] operationalised anticipatory socialisation using personal and educational factors (age, gender, school type and location, internship experience), whereas organisational socialisation was measured through first-job characteristics such as working months, unit placement, number of preceptors, and person–environment fit. Retention was treated as the key socialisation outcome.

Herzberg et al.’s [10,18] motivation-hygiene theory offers a complementary explanatory framework for understanding new graduate nurses’ responses to workplace characteristics during organisational socialisation. Motivators—including advancement, growth opportunities, recognition, and responsibility—contribute to job satisfaction, whereas hygiene factors, including interpersonal relationships, salary, supervision, and working conditions, are important for preventing job dissatisfaction.

## 3. Methods

### 3.1. Design and Data Source

This retrospective descriptive study aimed to examine how socialisation factors influenced early retention at the first hospital of employment. To ensure a representative sample of new graduate nurses across the country, this study utilised data from the Graduate Occupational Mobility Survey (GOMS), a government-approved secondary dataset recognised as official national statistics.

The sampling frame consists of graduates from all colleges and universities nationwide. GOMS employs a stratified multi-stage sampling design, with stratification first by academic department, followed by region and sex. Data are collected using a two-step process: initial contact is made by telephone to verify the sample list and confirm the participant’s willingness to respond; then, trained interviewers conduct face-to-face interviews either at the respondent’s home or workplace. Approximately 18,000 participants—representing about 4% of the national graduate population—are sampled each year. The final sample sizes were 18,081 in 2017, 18,163 in 2018, and 18,271 in 2019. Detailed information on the survey methodology and variable documentation is provided in the Graduate Occupational Mobility Survey USER GUIDE (2007GOMS–2019GOMS) [20]. The full dataset and codebook are available at: https://survey.keis.or.kr/goms/goms01.jsp (accessed on 1 August 2024).

### 3.2. Sample

The study population consisted of new graduate nurses who were employed at their first hospital after graduation from nursing school. Logistic regression analysis was performed using actual turnover among these nurses as the dependent variable. Based on a previous study indicating that a minimum sample size of at least 500 is required when performing logistic regression in large observational datasets [21], we ensured that the final sample size exceeded 500 participants.

A total of 896 nursing graduates were initially identified from the 2017–2019 GOMS datasets. Exclusion criteria were applied in the following order: graduates from advanced degree programmes or those who completed their studies outside the winter graduation period (*n* = 74), nurses not employed in hospital settings (*n* = 81), nurses with missing information on education or workplace (*n* = 115), and those aged 30 years or older (*n* = 24), who were considered statistical outliers exceeding the upper 5% of the age distribution. After applying these criteria, the final analytical sample consisted of 602 participants.

### 3.3. Measures

#### 3.3.1. Anticipatory Socialisation

Anticipatory socialisation consisted of personal and educational factors. Personal factors included age and sex. Educational factors included self-reported university GPA, internship programme participation, and location. Locations were categorised according to whether the participant’s high school, nursing school, and first hospital of employment as a nurse were located in the same or different regions.

#### 3.3.2. Organisational Socialisation

Organisational socialisation factors encompass workplace-related factors, motivation, and hygiene factors, based on the characteristics of the first hospital where the participants were employed. Workplace-related factors included hospital location (Seoul vs. other regions) and size (1000 or more employees and fewer than 1000 employees). Motivational and hygiene factors were assessed using single-item measures related to job satisfaction at the first hospital. Specifically, motivational factors included satisfaction with work itself, opportunities for career growth, and autonomy and authority over one’s work. Hygiene factors included dissatisfaction with interpersonal relationships in the workplace, salary, and working conditions.

In large-scale national surveys such as the GOMS, single-item measures are commonly used due to their efficiency and feasibility in large datasets [22]. Job satisfaction items—such as work itself, coworkers, and pay—have shown strong correlations with multi-item scales in previous studies, supporting their validity and reliability [23]. Based on this evidence, this study adopted single-item measures for the relevant constructs. Satisfaction-related items were rated on a 5-point Likert scale ranging from ‘very dissatisfied’ to ‘very satisfied’. Motivational factors were dichotomised into ‘satisfied’ (‘satisfied’ or ‘very satisfied’) and ‘not satisfied’ (including ‘neutral’, ‘dissatisfied’, or ‘very dissatisfied’), while hygiene factors were categorised into ‘dissatisfied’ (‘very dissatisfied’ or ‘dissatisfied’) and ‘not dissatisfied’. This categorisation method was based on previous research that treated Likert-scale job satisfaction items as categorical variables [24].

#### 3.3.3. Socialisation Outcome

Socialisation outcomes were defined as early retention at the first hospital of employment. In this study, early retention was defined as continued employment at the same hospital 18 months after graduation, at the time of the survey.

### 3.4. Analysis

Descriptive statistics (frequencies, percentages, means, and standard deviations) were used to summarise the characteristics of new graduate nurses in terms of anticipatory socialisation, organisational socialisation, and socialisation outcomes. Chi-square tests were conducted to examine bivariate associations between categorical independent variables and the dependent variable, hospital retention (coded as 1 for retention and 0 for turnover). Subsequently, multiple logistic regression analysis was performed to identify factors associated with hospital retention. Model fit was assessed using the Hosmer–Lemeshow goodness-of-fit test, and the model’s explanatory power was evaluated using Cox and Snell R^2^ and Nagelkerke R^2^ values. Results were reported as odds ratios (ORs) with 95% confidence intervals (CIs).

All independent variables were selected based on theoretical relevance, informed by the study’s conceptual framework, rather than through data-driven selection methods. All statistical analyses were conducted using SAS software, version 9.4 (SAS Institute Inc., Cary, NC, USA), and statistical significance was set at *p* < 0.05.

### 3.5. Ethical Considerations

This study involved a secondary analysis of publicly available, de-identified data obtained from a government-approved national dataset. As the dataset does not contain any personally identifiable information and no direct contact with human participants occurred, the study was deemed exempt from ethical review. The exemption was formally granted by the Institutional Review Board (IRB) of Yeungnam University College (IRB No. 2-7008156-AB-N-01-E-2024-001, Date of approval: 19 July 2024). According to institutional and national guidelines, research that utilises fully anonymised, secondary data without any intervention or interaction with human subjects is not subject to informed consent requirements. Therefore, informed consent was not applicable to this study.

## 4. Results

### 4.1. Retention Status of New Graduate Nurses

Table 1 presents participants’ retention status and employment duration at the first hospital. By September of the year following graduation, 68.6% remained employed at their first hospital, with an average tenure of 15.21 ± 3.85 months. Among all participants, 17.3% had transferred to another healthcare institution and continued working as nurses after working at their first hospital for an average of 5.57 ± 3.96 months. In addition, 1.8% were employed in non-nursing occupations, with an average of 6.36 ± 3.75 months at their first hospital. Lastly, 12.1% were unemployed at the time of the survey, although they had previously worked at their first hospital for an average of 8.58 ± 5.25 months.

### 4.2. Distribution of Socialisation Factors

Among anticipatory socialisation factors, the mean age was 24.35 ± 1.40 years (median = 24), and 83.7% of participants were female (Table 2). In terms of educational background, 60.8% had a GPA above B and 21.9% had participated in college-level internship programmes. Additionally, 28.9% of participants had completed high school, nursing school, and their first hospital all within the same region, suggesting potential regional continuity in their educational and employment pathways.

Regarding organisational socialisation factors, 29.4% of the participants were employed in hospitals located in Seoul, while 54.0% worked in hospitals with 1000 or more employees. Among the motivational factors, the highest satisfaction rate was reported for opportunities for career growth (47.8%), followed by satisfaction with the work itself (43.0%), and autonomy and authority over one’s own work (35.4%). Among the hygiene factors, the highest dissatisfaction rate was found for salary (26.2%), followed by interpersonal relationships in the workplace (23.3%), and working conditions (19.8%).

### 4.3. Socialisation Factors Associated with Retention

A chi-square test was conducted to examine associations between socialisation factors and hospital retention (Table 3). Among organisational socialisation variables, hospital location (χ^2^ = 14.23, *p* < 0.001) and hospital size (χ^2^ = 10.10, *p* = 0.002) were significantly associated with retention. Among the motivational factors, satisfaction with work itself (χ^2^ = 9.43, *p* = 0.002) and autonomy and authority over one’s own work (χ^2^ = 19.22, *p* < 0.001) showed statistically significant associations with hospital retention. Among the hygiene factors, dissatisfaction with interpersonal relationships (χ^2^ = 33.99, *p* < 0.001) was significantly associated with higher turnover. No anticipatory socialisation factors showed statistically significant associations.

A multiple logistic regression analysis was conducted to examine the factors associated with hospital retention (Table 4). The logistic regression model was statistically significant (χ^2^ = 89.45, *p* < 0.001). The Hosmer–Lemeshow goodness-of-fit test indicated that the model fit the data well (*p* = 0.618). The Cox and Snell R^2^ was 0.14, and the Nagelkerke R^2^ was 0.19. Among the anticipatory socialisation factors, having the same region for high school, nursing school, and first hospital was statistically significant, with participants in this category being 68% more likely to remain at their first hospital (CI: 1.09–2.60, *p* = 0.019). Among the organisational socialisation factors, nurses employed in hospitals in Seoul were 2.49 times more likely to remain than those employed in other regions (CI: 1.54–4.03, *p* < 0.001). Participants working in hospitals with 1000 or more employees were 2.21 times more likely to remain compared to those in hospitals with fewer than 1000 employees (CI: 1.47–3.34, *p* < 0.001). Regarding motivation factors, satisfaction with autonomy and authority over one’s own work was associated with a 153% increase in the odds of remaining at the first hospital (CI: 1.57–4.08, *p* < 0.001). Interestingly, satisfaction with opportunities for career growth was associated with a 41% decrease in the odds of retention, indicating a higher likelihood of turnover (CI: 0.39–0.90, *p* = 0.013). Finally, with respect to hygiene factors, dissatisfaction with interpersonal relationships in the workplace was associated with a 68% decrease in the odds of retention, corresponding to an increased likelihood of turnover (CI: 0.20–0.51, *p* < 0.001).

## 5. Discussion

This study analysed the retention status of new graduate nurses at their first place of employment 18 months after graduation, as well as the factors influencing this retention. The socialisation outcome examined in this study—the first hospital retention rate—was 68.6%, indicating that approximately 30% of nurses had left their initial positions. This figure is higher than the reported annual turnover rates for new graduate nurses, which range from 12% to 25% [9], and also exceeds the recent turnover rate of 21.0% among new nurses in Korea [19]. This discrepancy may be attributed to the longer follow-up period (18 months) used in this study, which includes nurses who left after more than 12 months of employment as well as cases of involuntary turnover. Nevertheless, the findings suggest that new graduate nurses experience significant challenges in adjusting to their first workplace, consistent with organisational socialisation theory and previous research.

Anticipatory socialisation, particularly geographic continuity between high school, nursing education, and the first hospital, was significantly associated with early retention. This continuity may facilitate the transition to professional practice by increasing familiarity with local healthcare systems, maintaining proximity to social support networks, and minimising relocation-related stress, thereby strengthening organisational commitment in the early career stage. Although this study did not directly measure social support, it is reasonable to infer that new graduate nurses working in geographically familiar regions may benefit from greater emotional and practical support from family and local networks. Prior research has likewise reported that such social support can buffer transition shock during the early stages of organisational socialisation and, in turn, reduce turnover intention [25]. Within this context, policies that promote regional continuity between nursing education and employment—for example, coordinated clinical placements within the same geographic area—may enhance new graduate nurses’ retention and contribute to the stability of the local healthcare workforce.

Organisational socialisation factors—particularly those aligned with Herzberg’s [10,18] motivation-hygiene theory—had a notable impact on early retention. Among these, satisfaction with autonomy and authority over one’s work and dissatisfaction with interpersonal relationships were consistently significant in both univariate and multivariate analyses. In particular, being accepted as a member of the nursing team is critical to early career retention, as it supports role adjustment and successful transition into the nursing role [26]. In the early stages of their careers, many new graduate nurses lack the competence to manage complex patient situations independently due to limited experience. A qualitative study also found that new graduate nurses often experience stress in handling their assigned responsibilities, which may contribute to early resignation [27]. However, when new graduate nurses develop competence and confidence in fulfilling their roles and are granted greater autonomy and authority [27,28], they are more likely to remain with their organisation. Furthermore, within the context of being accepted as team members, the findings of this study indicate that dissatisfaction with interpersonal relationships increases the likelihood of turnover among new graduate nurses. This aligns with previous evidence showing that positive relationships with colleagues, including managers, fellow nurses, and other healthcare staff, play a significant role in retention, and that supportive peer relationships can substantially reduce turnover among new graduate nurses [13].

Within the motivational factors of organisational socialisation, satisfaction with opportunities for career growth emerged as significant only in the multivariate models. Interestingly, this factor was associated with turnover rather than retention, suggesting a dynamic that contrasts with conventional assumptions regarding career development and employee retention. While career-related growth opportunities are generally believed to support employee retention, the findings suggest a pattern that departs from this prevailing understanding. Previous research has shown that career growth encompasses not only organisational advancement, such as promotion, but also professional development, including the enhancement of professional abilities [29]. Such professional development is closely linked to the experience of being accepted as a member of the nursing team and is strongly associated with managerial support and the provision of personalised educational programmes [26]. Thus, career growth opportunities may encompass not only the availability of advancement within the organisation but also the support systems that enable such opportunities. Without adequate support, even nurses who report satisfaction with growth opportunities may choose to leave for institutions that offer more supportive developmental environments. Moreover, recent evidence indicates that Generation Z nurses place greater emphasis on work–life balance and self-directed career development [30]. This may lead them to prioritise workplaces that align with their personal values over those that merely offer growth opportunities. In this study, the finding that 55.6% of nurses who left their first hospital subsequently re-entered the profession indicates that their departure did not represent an abandonment of the profession but rather a pursuit of organisations that provide more suitable support systems and are more consistent with their professional values. Taken together, these findings underscore the continued importance of offering growth opportunities that are accompanied by support mechanisms tailored to the preferences and developmental needs of younger nurses to more effectively address turnover concerns [31].

In this study, workplace-related factors of organisational socialisation, specifically the size and location of the hospital, were associated with the retention of new graduate nurses. Retention rates were higher in larger hospitals compared to smaller ones and in hospitals located in Seoul compared to those in other regions. Although this study did not directly assess organisational support systems, prior research has suggested that larger institutions are more likely to provide structured educational support, such as preceptorship programmes, which facilitate the adjustment of new graduate nurses to clinical practice and positively influence retention [32]. In line with these findings, the higher retention observed in Seoul may be partially explained by the concentration of large-scale hospitals in the capital, a trend consistent with previous studies reporting higher retention rates in urban compared to rural areas [33].

Based on the transition model, this study suggests that new graduate nurses’ successful adaptation to clinical settings is influenced not only by organisational resources but also by individual and social resources mobilised prior to entry into the workplace [34]. To support this transition effectively, new graduate nurses require structured support from healthcare institutions; encouragement from colleagues; and additional support from families, the community, and a familiar cultural environment. Collaboration between nursing education institutions and healthcare organisations is essential, and government support is imperative for its continuity and effectiveness [35].

### 5.1. Practical Implications

Sustained collaboration between nursing schools and hospitals is essential to reduce early turnover and support the clinical adaptation of new graduate nurses. Transition programmes, including structured orientation and mentoring, should be jointly developed and implemented by both institutions. Policy-level support is necessary to ensure the continuity and effectiveness of collaborative efforts. Nursing schools in regional areas should actively recruit students from local communities and establish systems that facilitate their transition to employment in nearby hospitals following graduation. Achieving the extended retention of new graduate nurses requires a multidimensional approach that includes emotional and cultural integration into the local community. Nurses who are familiar with the region are more likely to stay, as they may experience a stronger sense of belonging and comfort in their working and living environments. Supporting such community-based attachment through workplace and educational alignment can contribute to improving retention and workforce stability in underserved areas.

### 5.2. Strengths and Limitations

This study has several strengths. First, the use of secondary data from the GOMS provides a large, nationally representative sample of new graduate nurses. This enhances the generalisability of the findings within the Korean healthcare context. Second, unlike many prior studies that focus on intention to stay, this study examined actual retention at the first hospital of employment, offering a more accurate reflection of employment outcomes. Finally, the study was also grounded in organisational and anticipatory socialisation theory, which guided the selection and interpretation of variables.

Despite the abovementioned strengths, there are several limitations to consider. First, GOMS collects data approximately 18 months after graduation, requiring participants to retrospectively report their university and early work experiences, which introduces the risk of recall bias. Second, GPA was self-reported and may contain minor inaccuracies. Finally, since the study is based on data from Korean nursing graduates within a specific national and educational context, caution is warranted when generalising the findings to other countries or healthcare systems. Cross-cultural differences in educational pathways, workplace norms, and healthcare infrastructure may limit the applicability of these results beyond Korea.

## 6. Conclusions

This study examined factors influencing early retention of new graduate nurses at their first hospital of employment, using a conceptual lens of anticipatory and organisational socialisation. Findings related to anticipatory socialisation—such as regional alignment between nursing education and employment—highlight the importance of continuity in education-to-work transitions. Factors related to organisational socialisation, including hospital size, location, autonomy, interpersonal relationships, and growth opportunities, underscore the role of workplace environment in shaping retention. These results reinforce the relevance of socialisation theory in understanding early career outcomes and suggest that tailored strategies—such as regionally aligned placements and supportive work environments—may help improve nurse retention in the early stages of practice.

## Figures and Tables

**Table 1:** Retention status and duration of employment at the first hospital among new graduate nurses.

Retention Status	*n* (%)	Duration (Months), M ± SD
Retained at the first hospital	413 (68.6)	15.21 ± 3.85
Turned over		
Employed as a nurse elsewhere	105 (17.4)	5.57 ± 3.96
Employed in a non-nursing profession	11 (1.8)	6.36 ± 3.75
Currently unemployed	73 (12.1)	8.58 ± 5.25

Abbreviations: M: mean SD: standard deviation.

**Table 2:** Descriptive statistics of anticipatory and organisational socialisation factors.

Socialisation Factors	*n* (%) or M ± SD
Anticipatory socialisation factors	
Age	24.35 ± 1.40
Sex	
Female	504 (83.7)
Male	98 (16.3)
Grade point average	
Above B	366 (60.8)
B or lower	236 (39.2)
College internship participation	
Yes	132 (21.9)
No	470 (78.1)
Same region for high school, nursing school, and first hospital	
Yes	174 (28.9)
No	428 (71.1)
Organisational socialisation factors	
Hospital location	
Seoul	177 (29.4)
Other regions	425 (70.6)
Hospital size (number of employees)	
1000 or more	325 (54.0)
Fewer than 1000	277 (46.0)
Work itself	
Satisfaction	259 (43.0)
No satisfaction	343 (57.0)
Opportunities for career growth	
Satisfaction	288 (47.8)
No satisfaction	314 (52.2)
Autonomy and authority over one’s own work	
Satisfaction	213 (35.4)
No satisfaction	389 (64.6)
Interpersonal relationships in the workplace	
Dissatisfaction	140 (23.3)
No dissatisfaction	462 (76.7)
Salary	
Dissatisfaction	158 (26.2)
No dissatisfaction	444 (73.8)
Working conditions	
Dissatisfaction	119 (19.8)
No dissatisfaction	483 (80.2)

Abbreviations: M: mean SD: standard deviation.

**Table 3:** Univariate analysis of socialisation outcome by socialisation factors.

Factors	Retention	Turnover	χ^2^	*p*
Anticipatory socialisation factor				
Age				
22–24 (below median)	288 (69.7)	122 (64.6)	1.60	0.205
25–29 (above median)	125 (30.3)	67 (35.4)		
Sex				
Female	347 (84.0)	157 (83.1)	0.09	0.769
Male	66 (16.0)	32 (16.9)		
Grade point average				
Above B	252 (61.0)	114 (60.3)	0.03	0.870
B or lower	161 (39.0)	75 (39.7)		
College internship participation				
Yes	88 (21.3)	44 (23.3)	0.29	0.587
No	325 (78.7)	145 (76.7)		
Same region for high school, nursing school, and first hospital				
Yes	124 (30.0)	50 (26.5)	0.80	0.370
No	289 (70.0)	139 (73.5)		
Organisational socialisation				
Hospital Location				
Seoul	141 (34.1)	36 (19.0)	14.23	<0.001
Other regions	272 (65.9)	153 (81.0)		
Hospital size (number of employees)				
1000 or more	241 (58.4)	84 (44.4)	10.10	0.002
Fewer than 1000	172 (41.6)	105 (55.6)		
Work itself				
Satisfaction	195 (47.2)	64 (33.9)	9.43	0.002
No satisfaction	218 (52.8)	125 (66.1)		
Opportunities for career growth				
Satisfaction	199 (48.2)	89 (47.1)	0.06	0.803
No satisfaction	214 (51.8)	100 (52.9)		
Autonomy and authority over one’s own work				
Satisfaction	170 (41.2)	43 (22.8)	19.22	<0.001
No satisfaction	243 (58.8)	146 (77.2)		
Interpersonal relationships in the workplace				
Dissatisfaction	68 (16.5)	72 (38.1)	33.99	<0.001
No dissatisfaction	345 (83.5)	117 (61.9)		
Salary				
Dissatisfaction	105 (25.4)	53 (28.0)	0.46	0.498
No dissatisfaction	308 (74.6)	136 (72.0)		
Working conditions				
Dissatisfaction	74 (17.9)	45 (23.8)	2.84	0.092
No dissatisfaction	339 (82.1)	144 (76.2)		

Abbreviations: χ^2^: Chi-square test.

**Table 4:** Multiple logistic regression analysis of socialisation factors associated with hospital retention.

Socialisation Factors	OR (95% CI)	*p*
Anticipatory socialisation factor		
Age: 22–24 (below median) [ref: 25–29 (above median)]	1.38 (0.84–2.29)	0.207
Sex: female [ref: male]	0.88 (0.47–1.67)	0.698
GPA: Above B [ref: B or lower]	0.78 (0.53–1.17)	0.232
College internship participation: participated [ref: not]	0.79 (0.50–1.25)	0.322
Same region for high school, nursing school, and first hospital: yes [ref: no]	1.68 (1.09–2.60)	0.019
Organisational socialisation		
Hospital location: Seoul [ref: other regions]	2.49 (1.54–4.03)	<0.001
Hospital size: 1000 or more employees [ref: fewer than 1000 employees]	2.21 (1.47–3.34)	<0.001
Satisfaction with work itself [ref: no]	1.56 (1.00–2.43)	0.051
Satisfaction with opportunities for career growth [ref: no]	0.59 (0.39–0.90)	0.013
Satisfaction with autonomy and authority over one’s own work [ref: no]	2.53 (1.57–4.08)	<0.001
Dissatisfaction with interpersonal relationships in the workplace [ref: no]	0.32 (0.20–0.51)	<0.001
Dissatisfaction with salary [ref: no]	1.27 (0.81–2.00)	0.295
Dissatisfaction with working conditions [ref: no]	1.18 (0.71–1.96)	0.534
Likelihood ratio test χ^2^ = 89.45, *p* < 0.001, Cox and Snell R^2^ = 0.14, Nagelkerke R^2^ = 0.19		

Abbreviations: OR: Odds ratio, CI: confidence intervals.

## Data Availability

The dataset used in this study is publicly available from the Korea Employment Information Service (KEIS) through the KEIS survey website (https://survey.keis.or.kr, accessed on 1 August 2024). Researchers can access and download the data after completing the required registration process.
